# Anticipatory Care in Potentially Preventable Hospitalizations: Making Data Sense of Complex Health Journeys

**DOI:** 10.3389/fpubh.2018.00376

**Published:** 2019-01-28

**Authors:** Carmel M. Martin, Joachim P. Sturmberg, Keith Stockman, Narelle Hinkley, Donald Campbell

**Affiliations:** ^1^Monash Health Community, Clayton, VIC, Australia; ^2^University of Newcastle, Callaghan, NSW, Australia

**Keywords:** health trajectory, complex adaptive systems, potentially preventable hospitalizations, data analytics, anticipatory care, data science, frequent users, readmissions

## Abstract

**Purpose:** Potentially preventable hospitalizations (PPH) are minimized when adults (usually with multiple morbidities ± frailty) benefit from alternatives to emergency hospital use. A complex systems and anticipatory journey approach to PPH, the Patient Journey Record System (PaJR) is proposed.

**Application:** PaJR is a web-based service supporting ≥weekly telephone calls by trained lay Care Guides (CG) to individuals at risk of PPH. The Victorian HealthLinks Chronic Care algorithm provides case finding from hospital big data. Prediction algorithms on call data helps optimize emergency hospital use through adaptive and anticipatory care. MonashWatch deployment incorporating PaJR is conducted by Monash Health in its Dandenong urban catchment area, Victoria, Australia.

**Theory:** A Complex Adaptive Systems (CAS) framework underpins PaJR, and recognizes unique individual journeys, their dependence on historical and biopsychosocial influences, and difficult to predict tipping points. Rosen's modeling relationship and anticipation theory additionally informed the CAS framework with data sense-making and care delivery. PaJR uses perceptions of current and future health (interoception) through ongoing conversations to anticipate possible tipping points. This allows for possible timely intervention in trajectories in the biopsychosocial dimensions of patients as “particulars” in their unique trajectories.

**Evaluation:** Monash Watch is actively monitoring 272 of 376 intervention patients, with 195 controls over 22 months (ongoing). Trajectories of poor health (SRH) and anticipation of worse/uncertain health (AH), and CG concerns statistically shifted at a tipping point, 3 days before admission in the subset who experienced ≥1 acute admission. The −3 day point was generally consistent across age and gender. Three randomly selected case studies demonstrate the processes of anticipatory and reactive care. PaJR-supported services achieved higher than pre-set targets—consistent reduction in acute bed days (20–25%) vs. target 10% and high levels of patient satisfaction.

**Discussion:** Anticipatory care is an emerging trajectory data analytic approach that uses human sense-making as its core metric demonstrates improvements in processes and outcomes. Multiple sources can provide big data to inform trajectory care, however simple tailored data collections may prove effective if they embrace human interoception and anticipation. Admission risk may be addressed with a simple data collections including SRH, AH, and CG perceptions, where practical.

**Conclusion:** Anticipatory care, as operationalized through PaJR approaches applied in MonashWatch, demonstrates processes and outcomes that successfully ameliorate PPH.

## Introduction

Potentially preventable hospital emergency attendances or admissions occur as a consequence of the multiple domains influencing personal health journeys. Multimorbidity, frailty or systemic disease close to tipping points in personal health journeys are the main causes for the worldwide problem of rapidly rising rates of potentially preventable acute hospital (PPH) utilization (1, 2). Dynamics may encompass internal biological dysfunction promoting disease development or exacerbation; personal sense-making, strongly linked to feeling ill, having pain and/or experiencing anxiety and dysfunction. Local healthcare, social and living environments are significant influences. Big data analytics or other case finding methods can identify cohorts who are at risk of readmissions using a set of threshold parameters. However, understanding the complex systems of individual journeys in such cohorts in a timely manner requires an understanding of personal health and illness dynamics (3). Medicine is a human science that deals with the vagaries and uncertainties entailed in the multi-layered nature of “physical embodiment” within a multi-layered system encompassing ones' physical and sociocultural environment. This paper brings together theoretical frameworks from intellectual leaders and theory-informed empirical data from the Patient Journey Record System (PaJR) embedded in an ongoing, evolving and expanding clinical service- MonashWatch. In contrast with traditional epidemiological longitudinal studies with strict rules on time-fixed repeat observations, real world data systems that monitor and enable anticipatory and reactive care depend on pattern recognition in complex non-linear trajectories.

## Aims

In this paper, we make the case to utilize a complex adaptive system (CAS) framework to understand patterns in potentially preventable hospitalizations in adults using human support enabled by data analytics. Humans and human systems have been well-studied as CAS. While consensus does not exist on the nature of a CAS, five key characteristics capture major concepts identified in the literature: (a) diverse agents that learn and display choice, (b) non-linear interdependencies, (c) self-organization, (d) emergence, and (e) coevolution (4). Path dependence, historicity and retrospective coherence limit the ability to predict the future as the dynamics in CAS are non-linear and emergent. Nevertheless, anticipation and resilience emerge as observable patterns of CAS living systems, that are associated with survival in response to biopsychosocial challenges. In a previous publication, we proposed a pragmatic Theoretical Model for Static and Dynamic Indicators of Acute Admissions based upon an iterative and empirical analysis of data (3). This paper aims to provide a deeper explanation and analysis.

We propose an anticipatory care framework that incorporates resilience and interoception within an individual and cohort trajectory. We investigate data patterns in the PaJR MonashWatch deployment in order to demonstrate the utility of the theoretical framework in understanding.

### The Problem

Addressing potentially “preventable” emergency department utilization and emergency hospital admissions (PPH) in adults requires anticipation of unstable health journeys in a local network context. In the very short-term, problems need to be predicted in order to optimize hospital and community resources. Understandings of health have shaped this application of monitoring, responding and adapting to changes in health perception in vulnerable populations. Health professionals who work in challenging environments face competing demands of dealing with the needs of individuals and communities. They face constraints of time and available resources, and administrative impositions that limit necessary cross-boundary collaborations. Current research describes clinical approaches to cross-boundary patient care as “hit and miss” due to each member being contextually constrained by their own discipline (5). There are no easy solutions to these interconnected and interdependent problems which require system wide consideration (6) and how they can improve the care of vulnerable individuals identified as “frequent and sometimes preventable emergency hospital users.”

### The Patient Journey Record System (PaJR)

**Figure 1:**
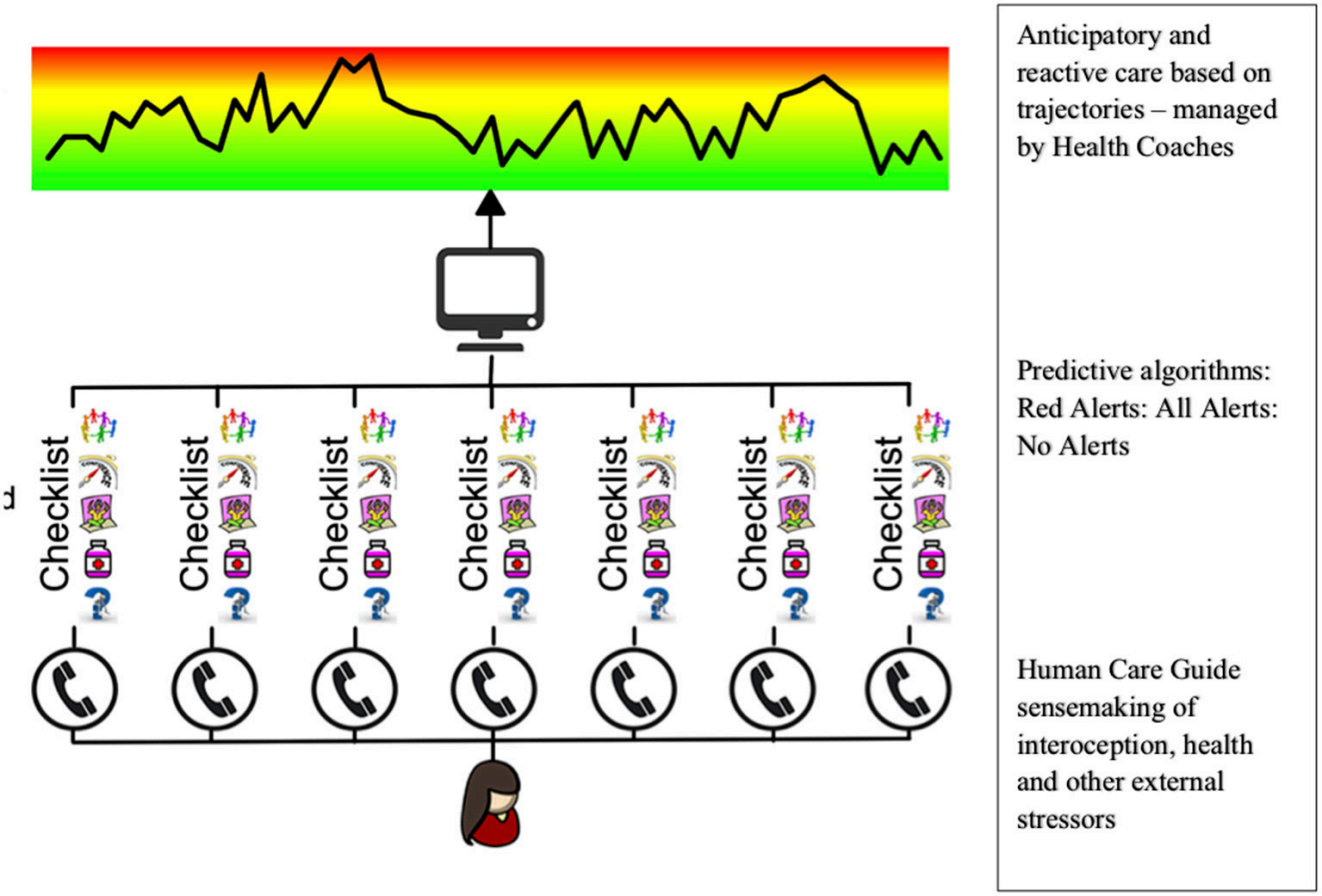
The structural design of the Patient Journey Record System (PaJR). Conversations form the core of predictive analysis analytics in real time. These conservations trigger alerts based on sense-making algorithms derived from conservations. Red alerts are those pertaining to medical and psychiatric symptoms of high risk and total alerts refer to both medical and biopsychosocial alerts relate to coping, self-care, social, and environmental issues. Lay callers from local communities are trained to use PaJR—Care Guides (CG). Alerts have been iteratively developed using ongoing adaptation and iterative learning and may be different in different settings (7).

The Patient Journey Record System (PaJR) has been developed to support individuals at risk of PPH. Regular conversations between staff and vulnerable individuals and their carers aim to make sense of their trajectories and anticipation of future directions of their health. These recorded conversations, as semi-structured data, create metrics to enable real-time decision making. This entails anticipating the changing dynamics of individuals, families and locations (hospital and home) in the context of diverse health and social care systems (see Figure 1).

**Figure 2:**
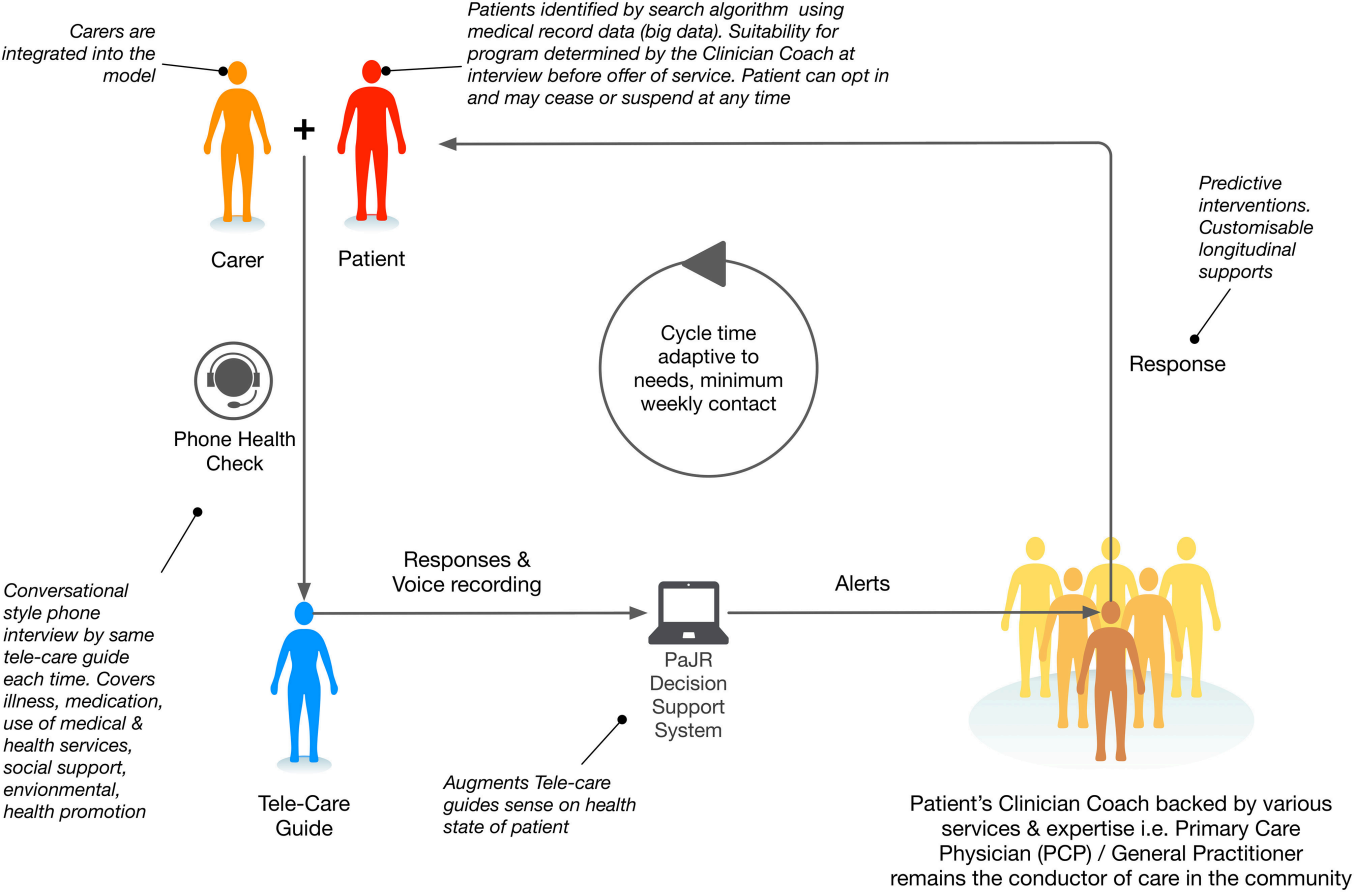
MonashWatch integrated care configuration incorporating the Patient Journey Record System PaJR.

PaJR applies a complex adaptive person-centered approach to understand and manage imminent deteriorations in health trajectories leading to potentially preventable hospitalizations. The system applies a *human sensing approach*—Care Guides (CG) regularly converse with “at-risk” individuals to track their concerns and self-perceived health (Figure 2). Health Coaches (nursing or allied health professionals) triage calls and supervise, visit patients at home, provide coaching and broker appropriate services where necessary.

The MonashWatch (MW) cohort consists of patients who are predicted to have ≥3 PPH/year based on hospital-based algorithms applied by the Victorian Department of Health and Human Services (DHHS)—the HealthLinks Chronic Care program (7).

### The Evaluation Data

The DHHS state-based public hospitals database HealthLinks Chronic Care (HLCC) utilizes clinical algorithms to predict a cohort at risk of ≥3 potentially avoidable hospitalizations (7). The HLCC algorithm identifies an eligible cohort of patients with general parameters including: unplanned admissions in past 6/12; ED visits in past 3/12; age; residence status, smoking; selected chronic conditions, such as digestive disorders, kidney disease, asthma, COPD, rheumatoid arthritis, diabetes, pancreatic conditions, cirrhosis/alcoholic hepatitis, and excluding conditions, such as cancer, dementia and serious mental illness. The DHHS supplies health services with a list of patients forming the “HLCC enrolled cohort” at the start of the trial and periodically updates lists based on ongoing analytics.

The Monash Health HLCC program called MonashWatch (MW) commenced its service in the Dandenong Hospital catchment, a low socio-economic status and ethnically diverse area of Melbourne (8). The PaJR system embedded in MW was developed in Ireland and validated in an Irish primary care cohort. The findings in this paper are based on an internal evaluation of the initial MW HLCC cohort in the intervention arm. Ethics approval was obtained from the Health Research Ethics Committee (HREC) of Monash Health for the conduct of the pilot service and its internal evaluation by the MonashWatch team.

### The PaJR MonashWatch Active Monitoring Cohort

MonashWatch currently actively monitors 272 of 376 intervention patients as the service is ongoing for 23 months, with new recruits and others dropping out due to the development of an ineligible condition, such as admission for psychosis, cancer or dementia, because of a death, improvement or admission to a nursing home, with 195 usual care controls. Because it is an ongoing program, patients become inactive with patients' death, admission to long term care, and other exclusion criteria including serious mental illness, cancer chemotherapy, renal dialysis etc. In addition, a person may drop out voluntarily or be transferred out to a more suitable program, also making them inactive. Thus, the cohort being monitored varies and individuals have a variable time period in the active intervention arm. Analysis is based upon the period in the program where there is intention to treat active patients. Outcome analysis is calculated by Monash Health and DHHS on the basis of observed verses predicted acute bed days costing of active patient exposure to the intervention. An external evaluation will be conducted utilizing intention to treat and other methodologies. However, the ongoing continuation and expansion of MW is primarily based on calculated cost savings in hospital over and above the cost of the MW service.

The participating MW cohort are all English-speaking but have very diverse ethnic backgrounds coming from Australia, Asian, West and East European, and Middle Eastern countries and elsewhere. The MW Care Guides and Health Coaches were all from the local community but did not mirror the diversity of the MW cohort.

The PaJR system generates alerts based on semi-structured data entered by CG. Alerts are based on clinical algorithms which are iteratively adapted. Details have been described previously (9), however a summary is provided. *Red alerts* are warning indicators that require clinical assessment either immediately, or within a specified time within 24 h, are generated by an automatic algorithm in PaJR. Features that trigger red alerts include chest pain, severe pain of any nature, a fall, a mental health crisis or housing crisis, and recent attendance at the ED. Red alerts reflect typical disease symptoms that are likely to lead to hospitalization. *All alerts* reflect the number of problems identified per person per call including coping, illness, health care services, medication social and environmental concerns (see Figure 2).

## Emerging Theoretical Frameworks

A Theoretical Model for Static and Dynamic Indicators of Acute Admissions (TSDIAA) (3) was derived iteratively and empirically during the initial 6/12 of PaJR MonashWatch service deployment using additional measures over and above the HLCC original service and disease predictors of repeat admissions. TSDIAA additional baseline measures included Clinical Frailty Index (CFI) (10); Connor Davis Resilience (CD-RISC) (11): SF-12v2 Health Survey scores Mental (MSC) and Physical (PSC) and ICEpop CAPability measure for Older people (ICECAP-O). PaJR call data and acute (non-surgical) admissions from Victorian Admitted Episode database (3). The theory was that less systemic resilience (CFI); and lower (CD-RISC) would be associated with worse mental, physical health and quality of life (SF-12v2 Health Survey scores Mental (MSC) and Physical (PSC) and ICECAP-O. These measures would influence or even predict PaJR alerts per call and acute admissions.

The TSDIAA measures were significantly intercorrelated except mental health (SF-12_MCS). SF12-MSC, SF12-PSC, and ICECAP-O best predicted PaJR alerts/call (ROC: 0.84). CFI best predicted acute admissions (ROC: 0.66), adding CD-RISC, SF-12_MCS, SF-12_PCS, and ICECAP-O with two-way interactions improved model (ROC: 0.70). Thus, psychosocial resilience and frailty, and mental, physical health and quality of life were influential on alerts, while physical frailty was most influential on actual admissions. Nevertheless, dynamic indicators are needed to determine when and how and possibly why the alerts, as proxies for biopsychosocial stressors and admissions took place.

## Transition From Theory to Practice

This section explores more in-depth theory and analysis related to complex adaptive systems and predicting or anticipating patterns in unstable journeys. Precise prediction of hospital admissions in non-linear, dynamic complex health systems, at present, remains challenging despite ongoing improvements in modeling (12).

Complex adaptive systems (CAS) describe the interconnected and interdependent nature of trajectories. CAS exhibit many to many relationships that typically create feedback loops which create dynamic behavior. Changes to the configuration and/or relationships within the trajectory alter its behaviors and its observable characteristics (or outcomes) in almost unpredictable ways (only very short term predictions); so that unanticipated or irregularly timed events emerge in the unstable journeys.

Features of the CAS system applicable to potentially preventable hospitalizations (13) include:

- Individuality: individual journeys are subject to and driven by decentralized, local interactions of constituent parts and personal sense making.
- Historicity and path dependence; the path is always dependent on its history and predicting the future is challenging; thus there is a need for models about prediction and anticipation.
- Agents that learn: learning is understood in terms of the adaptive behaviors of patients and their caregivers. Clinical teams can also be viewed as agents that learn—agents will learn according to how they are constrained internally or externally.
- Heterogeneity: substantial diversity in the dynamics of aging, illness and dying, and society's responses.
- Feedback: a CAS usually contains many interdependent interacting pieces, connected across different levels. System dynamics are often characterized by feedback and substantial non-linearity.
- Emergence: personal narratives and sense-making emerges from an individuals interoception about their internal state of health and an external environment and context.
- Unintended consequences commonly emerge (well-intentioned acute medical care may result in fruitless investigations, loneliness, and hospital acquired infection or post-hospital syndrome).
- “Tipping points”: non-linearity means that the impacts caused by small changes can seem hugely out of proportion. The individual may spend long periods in a state of relative stability yet be easily “tipped” to an avoidable hospitalization by a disturbance in one of many domains—including biopsychosocial and environmental—that pushes illness and social support across an individual threshold.

**Figure 3:**
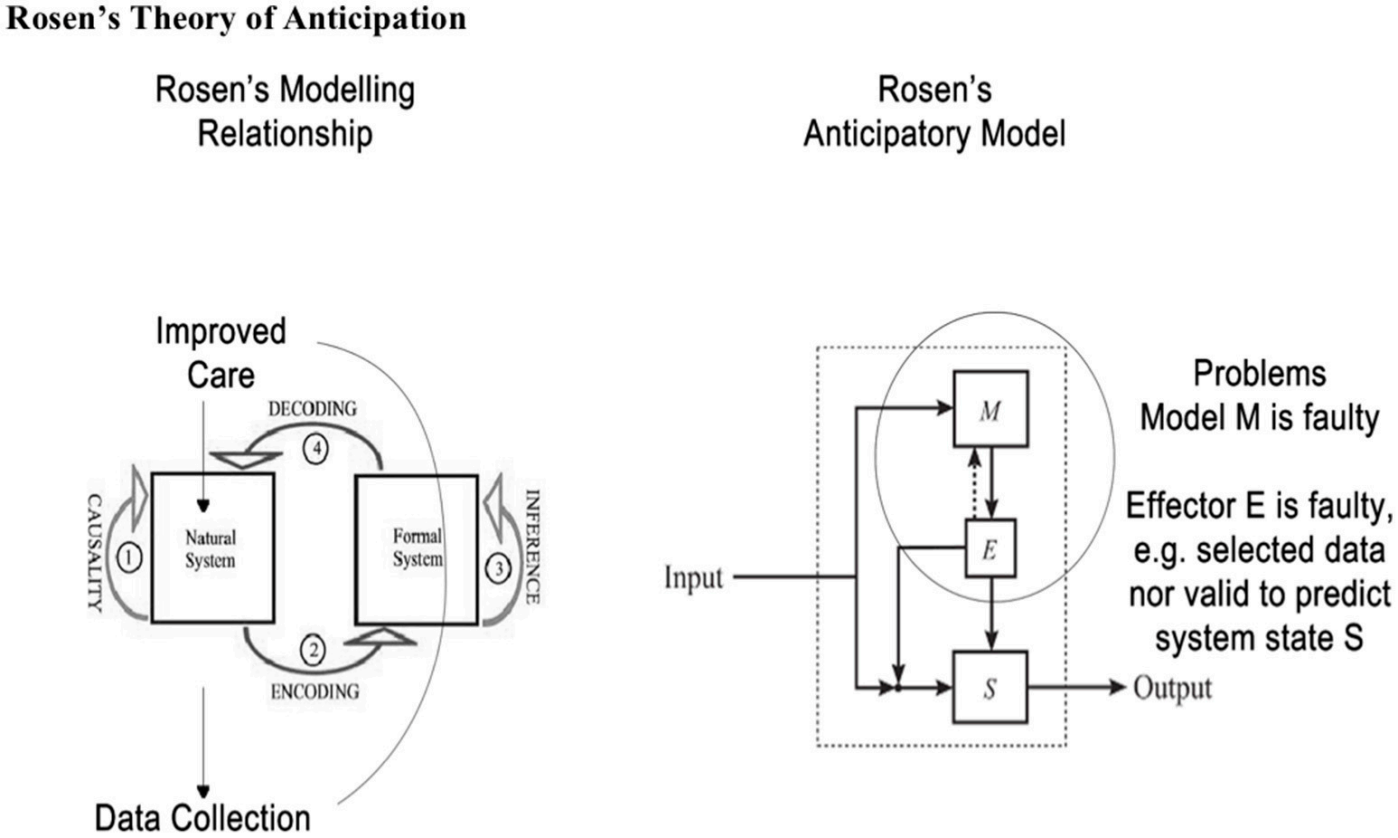
Rosen's modeling relationship and the potential for modeling errors (9, 10).

In this paper we also draw upon Rosen's insights to understand further nature of anticipation or prediction and adjustment to improve outcomes in the dynamics of unstable health journeys. In general, Rosen's Modeling Relation is a way to compare unstable trajectories of what is observed compared with anticipated trajectories in a human journey. That is at every journey point there is state (present) and a (very short term) prediction of a future state. The closer the modeling relation is to the real world the better the prediction and hence actions taken by the anticipatory system (MonashWatch) (see Figure 3).

Anticipation, in Rosen's work, implies that one or more prediction models result in an adjustment of present behavior in order to address an anticipated future situation (desirable or undesirable). In other words, an anticipatory entity (system or whatever) takes its decisions in the present according to forecasts about something that may eventually happen. The best-known definition of anticipation is Rosen's: “An anticipatory system is a system containing a predictive model of itself and/or its environment, which allows it to change state at an instant (or time point) in accord with the model's predictions pertaining to a later instant” (14).

Anticipation in Rosen's view is a style of control which differs from goal oriented feedback control. System behavior is anticipatory when the system (S—a natural system) possess a predictive model (M—a formal system) of itself and/or its environment which via effector(s) (E) modifies the behavior of S in light of M's prediction. Anticipatory behavior is ubiquitous in living systems e.g., ducking before something thrown at you hits you. The future event of being hit is anticipated—the prediction is not perfect but useful in this situation. Flaws in the modeling relationship may lead to undesirable or lack of adaptive behavior as M or E is faulty. Rosen's model provides a useful frame for health care which anticipates as well as reacts, especially when it leverages both patients' and carers' anticipatory behaviors and augments it with clinician decision support models like PaJR. We have barely scratched the surface of Rosen's insights here.

### Complex and Ill-Defined Demand in Frequent Users of Emergency Hospital Care

Modern emergency departments (ED) and acute non-surgical wards are struggling to cope with complex and ill-defined demand. This is shaped by social and non-social determinants of health, and the community-based health and social system's constrained responsiveness.

A significant proportion of admissions for complex and ill-defined conditions cause actual harm and/or do not necessarily improve individual health journeys (15, 16). These observations open up both ethical and philosophical questions:

- How can we predict when people are likely to have an acute potentially preventable hospitalization (tipping points)?
- How can we anticipate actions needed to change trajectories in order to optimize hospital admission?

There is a plethora of work in the literature that deals with developing (re)admission risk prediction models, but most of them do not have sufficient utility to be deployed as a stand-alone in a clinical setting. They fail in part because they neglect to account for local environments being unique complex adaptive systems, and that hospitals have a fuzzy rationale for their existence (17). Nevertheless, as discussed previously the timing and nature of readmission—even when anticipated—are unpredictable (16).

### Anticipating Tipping Points in Journeys in PPH

Personal data analytics in a complex adaptive system framework uses a form of anticipatory trajectory modeling (18). Early warning systems to identify health deterioration are gaining scientific rigor and currency in healthcare, particularly in critical and frailty care (19). Potential solutions, that all can predict death, dependency, and disability in specific circumstances, include:

1. Biomarkers, biometrics, and patient-reported outcome measures (20)
2. Measures of personal health experiences and quality of daily life (21)
3. Technological solutions like smartphone applications and self-monitoring sensor devices for active or passive monitoring (22)
4. Interoception approaches are manifested by the conversation between the body and brain via multiple afferent and efferent feedback loops. Listening in on this process requires different approaches that include many of above (1, 2 & 3) approaches within a framework including self-rated health, voice prosody and general vitality that human carers (23) and care providers can identify through ongoing conversations.

Very vulnerable individuals who are most at risk of hospitalization may have triggers which are not necessarily related only to specific disease process or psychosocial or environmental crises but a “gestalt of events” in individuals with low resilience (24). It is very difficult for standard “objective” metrics to ascertain the meaning of a situation, or conversely, the situatedness of meaning of a potential health crisis (25, 26). These patients will likely *sense* an imminent crisis, due to the nature of human interoception and sense-making associated with survival (27).

### Operationalizing Anticipation Through Interoception and Sensemaking in Trajectory Analytics

Humans appear to have—at a deep intuitive level—evolved “the capacity to *know* their physiology and its trajectory” that otherwise is difficult to categorize (21, 28)—a phenomenon known as interoception. Our human capacity for interoception is reflected in our “*self-rated health* perceptions” (29). *Self-rated health* appears to account for physiology, meaning and situatedness, and has predictive power related to anticipating mortality, hospital admission, and service utilization (21). Regular conversations may tap into interoception of personal health states (*self-rated health*) but also provide information about the meaning and chronology of the experiences so that potential crises might be averted. Conversations and narrative-based support over time help people make sense of their interoception in the context of their health journey (30, 31), and at the physiological level result from modulation of psychoneuroimmunology pathways (32).

### Trajectories

Hollar notes*: “Trajectory analysis in health care involves the mapping of sequences of events and multiple variables contributing to health outcomes for individuals. As such, the term ‘trajectory' is used in a stochastic, approximate sense, because we can never perfectly predict the future for any process”* (18). Historically, Poincare, Prigogine, Ruelle, Thom, Rosen, and other complexity scientists have demonstrated a sensitive dependence on initial conditions in any complex system. Furthermore, healthcare decision-making in the context of multi-dimensional biopsychosocial and environmental stressors can be inappropriate based on monitoring the “wrong” frames or using linear and/or superficial assumptions when predicting the nature of journeys. Trajectory analytics link closely with other scientific theories, interoception (29)—capacity of the body to anticipate its trajectory and systemic resilience (33)—ability to bounce back upon disease, physical and other challenges, ultimately determine the chances of survival. The Embodied Predictive Interoception Coding model, for example, integrates an anatomical model of corticocortical connections with Bayesian active inference principles, to propose that cortices contribute to interoception by issuing interoceptive predictions based on feedback on the current bodily state and predictions (30, 34).

Anticipatory care for people with complex trajectories should monitor perceived health and needs that emerge from the dynamic network interactions between the microlevel of individual biology to the macrolevel factors of their environments. Clinical team functioning will be dependent upon the usefulness of their models or representations in the present environment and the future environment (35). Anticipatory care is often supported by trajectory data analytics with expanding opportunities for predictive analytics and learning how to apply anticipatory principles to improve outcomes (36).

### PaJR Applies Anticipation, Interoception, and Trajectory Models in a CAS

In the design of the PaJR system, the central personal narrative revolves around the current state of health problems and stressors and an anticipated future state over the next few days. Using clinical algorithms, a risk score based on aforementioned alerts indicating wellness, a highly acute or a less acute but potentially problematic state based on interoception and background reports (37). The original state of the person when embarking on the journey sets the trajectory, for example frailty, at baseline is the major predictor of admission in the Monash Watch cohort above the threshold of previous high levels of readmissions and emergency department use (3, 21).

The PaJR system applies a human sensing approach—Care Guides (CG) who regularly converse with “at-risk” individuals to track their concerns and self-perceived health (Figure 2). Understandings of health have shaped this monitoring and responding to changes in health perception in vulnerable populations. Health Coaches supervise and triage the CG calls, providing direct responses and recommendations, such as visits to primary care or urgent transfer to hospital and anticipatory care to ameliorate future tipping points, such as medication, health care of social/welfare and environmental interventions. This demonstrates the involvement of carers and the broader systemic implementation. Care Guides call the patient and/or carer at least weekly, to conduct a health check in the from of a semi-structured conversation which is encoded into PaJR as point data and paraphrased narrative. PaJR decision support algorithms then generate health decline risk alerts. The Health Coach (allied health and nursing) respond through an initial triage, possible home visit and activating local service networks. Rosen's model provides a useful frame for PaJR which anticipates as well as reacts, especially when leveraging both patients' and carers' anticipatory behaviors and clinician decision support.

## Evaluation—Process and Impact, and Acceptability of PAJR-Supported Anticipatory Care

### Anticipating and Ameliorating Potential Tipping Points in the Patient Journey

Does an anticipatory approach in MonashWatch work? So, we start from the idea of anticipation - the idea that there is more than one scale of real time in any health trajectory. Various time-lines are tied to each journey, with the multiple domains exhibiting dynamic trajectories in what we recognize to be a complex system. Hence we search for patterns in the resultant complex dynamics of the MW cohort which might signify an anticipatory time-line.

Three measures of anticipation were selected from the regular call data base in the 7 days before an acute non-surgical admission to see if a tipping point pattern would emerge, that might allow adaptation of care in some manner. Then individual trajectories are described to demonstrate anticipatory activities and their results in 3 randomly selected cases.

The data set is described in Table 1.

**Table 1:** Process and outcomes of Internal evaluation of Monash Watch service pilots (as of 25/10/2018).

**Cohort**	**Monashwatch**
Case finding	Health Links Chronic Care Algorithm (7)
Setting	Monash Health Dandenong Hospital Catchment (city)
No of participants	currently actively monitoring 272 of a total of 376 Pilot Intervention patients, 195 controls
Time	22 months (ongoing)
Number of phone calls to date	15,627
Age (Median)	75 (31–91)
Gender	55% female
Alerts per call (Problems identified)	Median 1, mean 2.7.
Red (clinical-type) alerts/call requiring prompt attention	Median 0. mean 0.3
Most common “symptoms of concern” reported	Infection, pain, unsteady, collapse, weak, falls, depression, mental health, cough, wheeze
Primary targets	Reduction of emergency hospital bed days by >20% vs. in intervention vs. controls *p* < 0.05. Target reduction 10% which was significantly exceeded

### Trajectories

Time series analysis were based on 303 calls, that took place 7 days prior to an admission. Calls were to 103 MW patients (who were admitted as an emergency admission of >1 day) and took place within 6 months of the start of MW program. An analysis of both self-rated health (SRH) at the time of the call which incorporates interoception, and anticipated health (AH) over the next few days (anticipating/expecting uncertain or worse health vs. improved or same health) and CG concerns about the patients' were analyzed. At the end of each call the CG was asked to rate whether they were concerned about this patient based on voice, tone, breathing and prosody, as much as what was said, based on their knowledge of how the patient usually was.

### Identifying Tipping Point Patterns in Trajectories

#### Methods

The initial stages in the analysis of a time series may involve plotting values against time to examine homogeneity of the series in various ways: stability across time as opposed to a trend; stability of local fluctuations over time. Irregular times series present a challenge to current time series analysis which assume regular time series. Thus, exploratory data analysis (EDA) is an approach to analyzing data sets to summarize their main characteristics, often with visual methods. Homogeneity measures using Pettitt's model is a method of describing the central location of the pattern being observed.

Homogeneity tests involve a large number of tests for which the null hypothesis is that a time series is homogenous between two given times. The variety of the tests comes from the fact that there are many possible alternative hypotheses: change in distribution, changes in average (one or more times) or presence of trend. The Pettitt's test was selected as it is a non-parametric test that requires no assumption about the distribution of data. The Pettitt's test is an adaptation of the rank-based Mann-Whitney test that allows identifying the time at which the shift occurs. The tests correspond to the alternative hypothesis of a single shift. For this test, XLSTAT provides *p*-values using Monte Carlo resampling.

**Figure 4:**
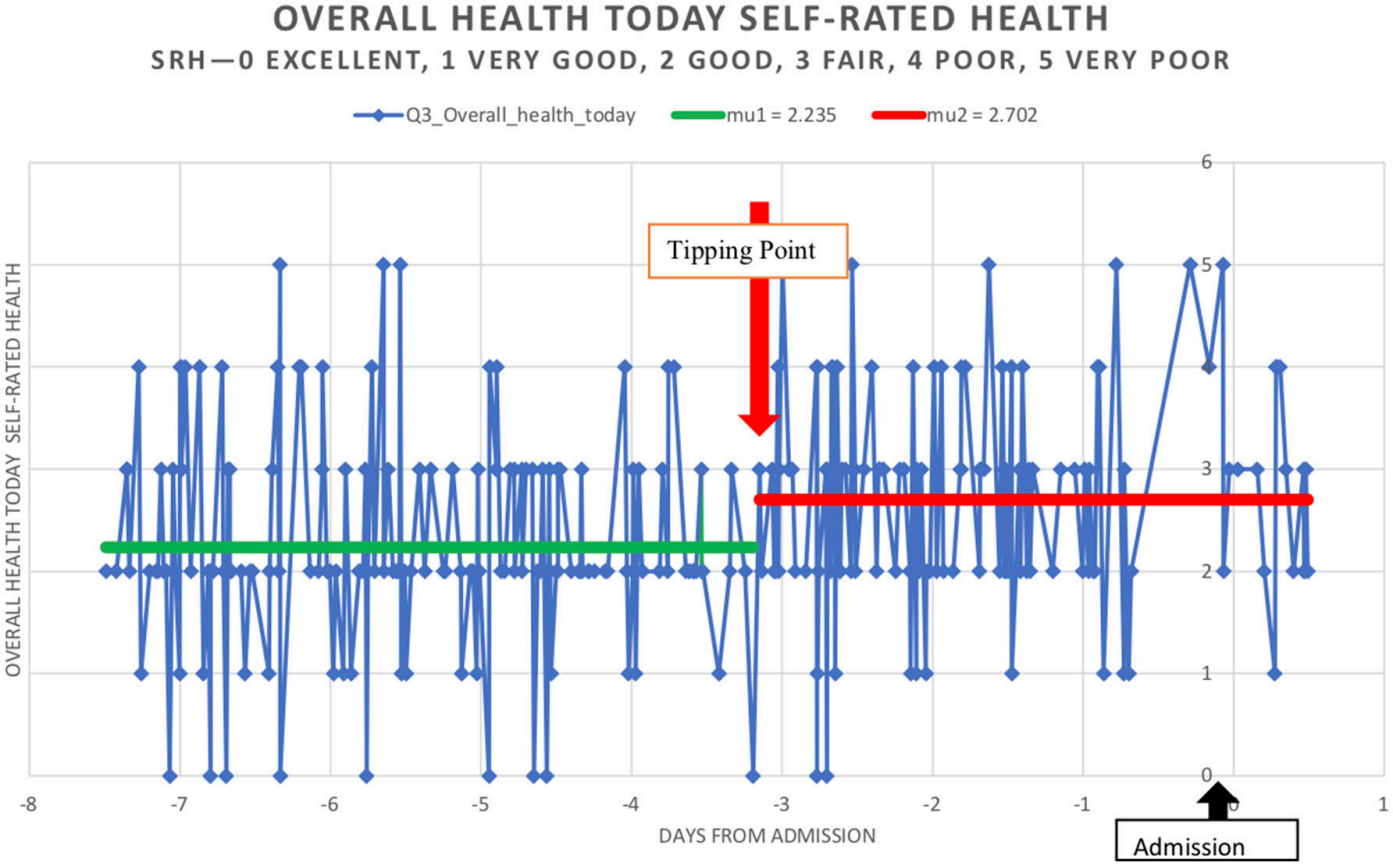
Trajectories of overall health today [Self-Related Health' (SRH−0 excellent, 1 very good, 2 good, 3 fair, 4 poor, 5 very poor)] in 303 calls, that took place 7 days prior to an admission. Calls were to 103 MW patients (who were admitted as an emergency admission of >1 day) and took place within 6 months of the start of MW program.

Figure 4 and Table 2 describe how personal health perceptions human health anticipation or interoception in care trajectories shifted before acute admissions. The CG mirrored the patients “anticipation” of potential admissions around day 3—a tipping point—before an admission.

**Table 2:** Pettit's test of homogeneity in MonashWatch (MW) trajectories of overall health today (Self-Rated Health' (SRH−0 excellent, 1 very good, 2 good, 3 fair, 4 poor, 5 very poor) and Anticipated Health over next few days (0 improve/stay same; 1 uncertain/get worse) in patient journeys before admission and Care Guide concerns (0 = no, 1 = yes).

**Pettitt's test**	**Self-Rated Health' (SRH−0 excellent, 1 very good, 2 good, 3 fair, 4 poor, 5 very poor)**	**(Anticipated health over the next few days): (0 improve/stay same; 1 uncertain/get worse)**	**Care guide concerns (0 yes; 1 no)**
All MW cohort *N* = 303 calls	0–5	0–1	0–1
Median (mean; std)	2 (2.34 ± 1.05)	1 (0.53 ± 0.5)	1 (0.67 ± 0.46)
*K*	2,458	5,160	4,203
*T* (day of “tipping”)	−3	−3	−3
*p*-value (two-tailed) confidence intervals	0.03 CI [0.028, 0.037]	0.001 CI [0.000, −0.002]	0.004 [0.003, −0.006]
alpha	0.05	0.05	0.05
**75+ (*****n*** **= 142 calls)**			
*K*	1,396.000	408.000	1,014.000
*T* (day of “tipping”)	−3	−3	−3
*p*-value (two-tailed) confidence intervals	0.021 [0.017, 0.025]	0.821 [0.811, 0.831]	0.092 [0.085, 0.10]
**< 75 (*****n*** **= 148 calls)**			
*K*	1,442.000	906.000	1,146.000
*T* (day of “tipping”)	−3	−4	−2
*p*-value (two-tailed) confidence intervals	0.044 [0.039, 0.049]	0.066 [0.059, 0.072]	0.069 [0.063, 0.076]
**Male (*****n*** **= 173 calls)**			
*K*	1,911.000	915.00	1,878.000
*T* (day of “tipping”)	−3	−4	−4
*p*-value (two-tailed) confidence intervals	0.023 [0.019, 0.027]	0.158] [0.148, 0.167]	0.003 [0.002, 0.005]
**Female (*****n*** **= 114 calls)**			
*K*	820.000	450.000	486.000
*T* (day of “tipping”)	−3	−3	−4
*p*-value (two-tailed) confidence intervals	0.195 [0.185, 0.205]	0.33 [0.318, 0.343]	0.289 [0.277, 0.300]
alpha	0.05	0.05	0.05

*Day of admission is 0*.

*The p-value has been computed using 10,000 Monte Carlo simulations*.

A tipping point was identified at 3 days prior to an acute admission in the 103 patients as a group with the shift to worse health being statistically significant for SRH, AH and CG concern on day −3. Figure 4 describes the pattern shift or tipping point of SRH today. Subgroup analysis—males and females and age groups < 75 and 75+ confirmed these patterns although the numbers were small. SRH showed a significant shift to worse health on day −3 in males and < 75 and 75+ was not statistically significant in females although the trend was present. The same trend, which was not significant, existed in AH and CG concerns for females. However, CG concerns tipping to worse on day −3 were significant for males.

#### Case Studies

**Figure 5:**
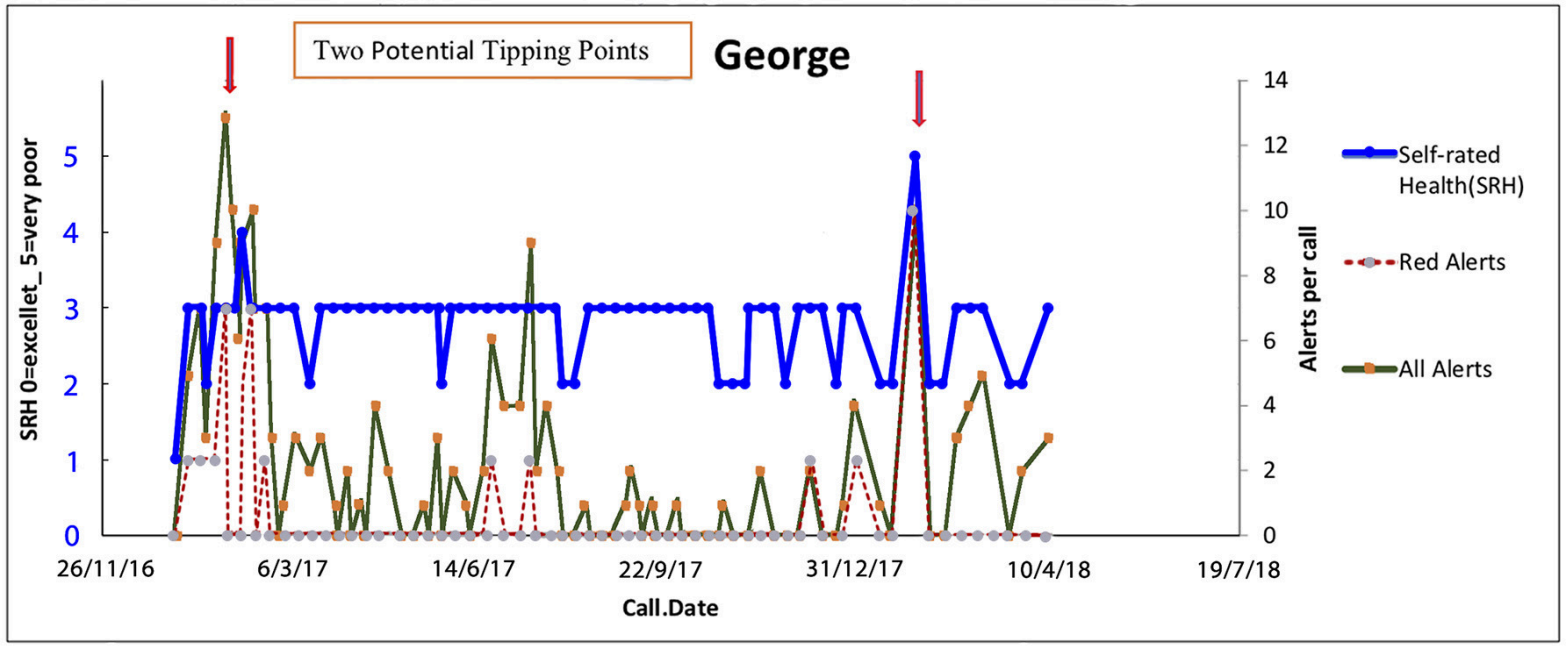
Tipping points in George's health journey on 15/1/17 with no subsequent acute admissions. Trajectories of overall health today [Self-Related Health' (SRH−0 excellent, 1 very good, 2 good, 3 fair, 4 poor, 5 very poor). Red alerts are those pertaining to medical illness symptoms and total alerts refer to both medical and all alerts relate to wider biopsychosocial issues including coping, self-care, social, and environmental issues].

**Figure 6:**
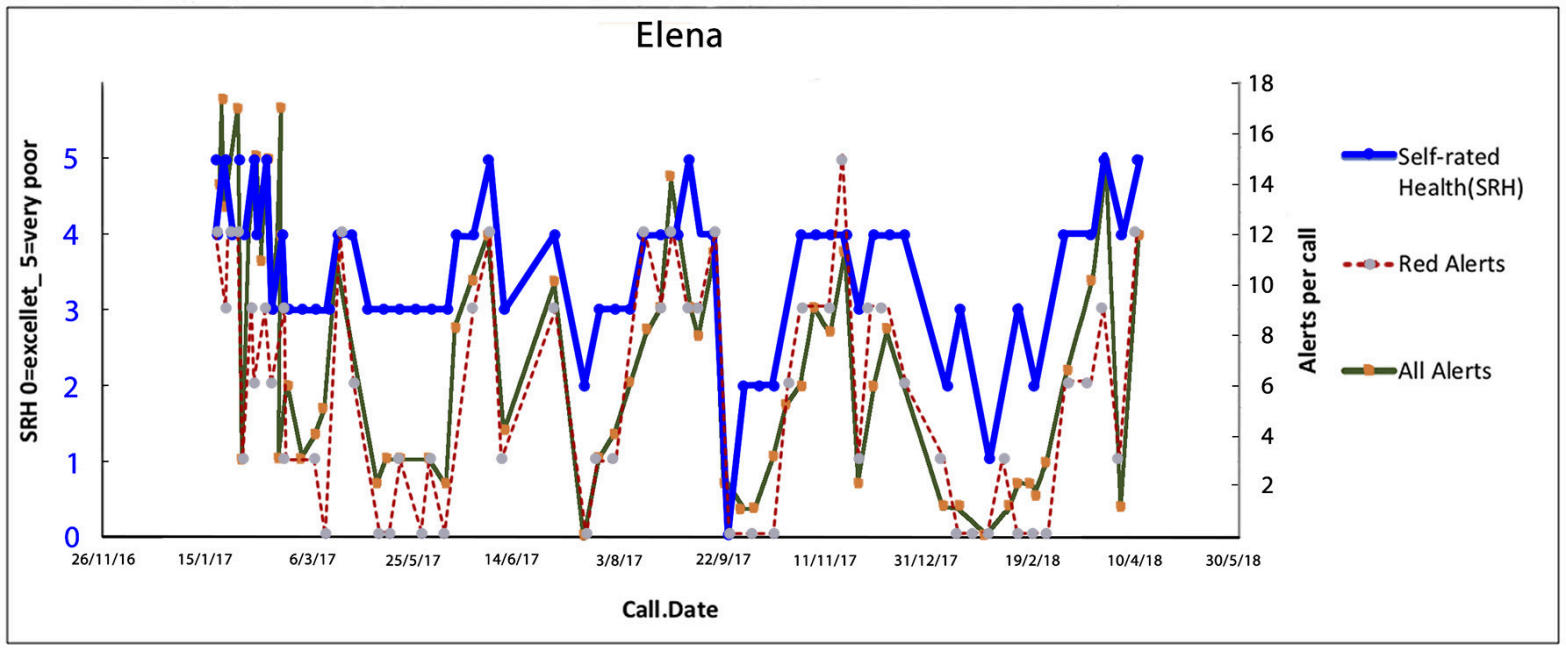
Elena's trajectory through illness triggered by financial crisis, legal issues, the social isolation of care for her children and managing her hospital treatments. Multiple trigging points in Elena's health (biopsychosocial) journey with subsequent acute admissions while in the MW service. Trajectories of overall health today [Self-Related Health' (SRH−0 excellent, 1 very good, 2 good, 3 fair, 4 poor, 5 very poor). Red alerts are those pertaining to medical illness symptoms and total alerts refer to both medical and all alerts relate to wider biopsychosocial issues including coping, self-care, social, and environmental issues].

Tipping points in patient trajectories are anticipated and ameliorated, if possible, through prompt attention by appropriate services, or if not anticipated or ameliorated, tracked in order to learn about how best to improve that individual's journey. The following case studies are real trajectories, but patient names and some details have been modified to ensure that the patients are non-identifiable. Australian date formats (dd/mm/yy) are used below and in the accompanying figures (5, 6 & 8).

#### George—An Acute Health Crisis

George (a pseudonym) is 81 years of age, and a widower, whose self-rated health had a gradual linear trend to improvement over the past 3 months since receiving MonashWatch services. George lives alone with his several pets. His neighbors are also elderly, and none of his family lives near-by. He spends a lot of time in his garden, the pride of his late life. On 14/1/17, during a heat wave, he experienced acute illness symptoms and had great difficulty managing without air conditioning at home. During the phone call on 15/1/17 with the CG, George sounded weak and reported worsening self-rated health and many concerns about his environment, and he was most concerned about his pets. He declined to go to hospital. The Health Coach (nursing or allied health professional) promptly addressed this potential admission with a home visit, and guided by a hospital physician, rehydrated George in the home, and organized home-care services, an occupational therapy home safety assessment and a GP home visit (Figure 5).

The key anticipatory triggers in George's trajectory on 15/1/17 are George's intrinsic self-knowledge that his journey could be managed at home *t* + 1, although at *t* + 1 he knew he was in a bad way—his self-rated health dropped from good to fair. His situation at home with his pets was highly distressing for him. The assessment by the team that while at *t* + 1 his state of health was very poor, there was an intervention that could rescue George.

On 14/7/17 George had another crisis, but his anticipation that he could stay at home allowed him to report his infection to the attention of the CG early enough for the Health Coach to intervene.

#### Elena—Repeated Environmental Stressors as Triggers of Physical Symptoms

Elena (a pseudonym) is the mother of children with disabilities, as well as having financial debt, social isolation and food security issues. She also has multiple morbidities including migraines, severe hypertension and chronic pain. She has recently lost the main social support person in her life. Each of the peaks represent financial and environmental crises related to managing her children's well-being which triggered headaches, hypertension and pain. Elena was well-known to multiple services—housing, child health, social welfare, the emergency department, endocrinology, pain clinic, psychological medicine, and various charities. Since her recent bereavement, she had exhausted many relationships with her family/community, and with helping professions because she defaulted appointments and found difficulty complying with spending restrictions, and recommendations by social welfare, and attending her hospital appointments. However, central to all her activities was the care of her children (Figure 6).

The Health Coach and the CG initially formed a supportive relationship providing a “friendly” therapeutic alliance to enable Elena to reduce her crises and panic episodes. The Health Coach visited Elena at home conducting a “deep dive” to identify the dynamics behind each panic attack. The team then worked with Elena to find crisis solutions which included vouchers to obtain food from a charity, taxi vouchers to travel to appointments, a medication review by her GP to optimize drug treatments and minimize her drug-related side effects of nausea, hand, and ankle swelling, rearranged hospital appointments to allow Elena to prepare her children for school etc. Over time longer term solutions included training related to budgeting including strict monitoring of her financial situation; self-management training to address panic attacks, close liaison with her GP and hospital endocrinology team, and accompanying Elena for invasive tests. Crises were continually detected through the ongoing CG calls. Ongoing informational appraisal, and practical and peer support allowed her to build connections with her local community. These diverse interventions have been gradually stabilizing Elena's journey.

The key anticipatory triggers in Elena's trajectory were related to running out of money and inability to feed her disabled children and her severe pain symptoms. Elena, on each anticipated tipping point occasion, was visited by her Health Coach to assist with coping strategies and ensure appropriate services were in place. Elena never attended the ED and/or was admitted during the whole year in the program. Through phone calls, and home visits the team took a non-judgmental approach working to support Elena to regain some control over her life.

**Figure 7:**
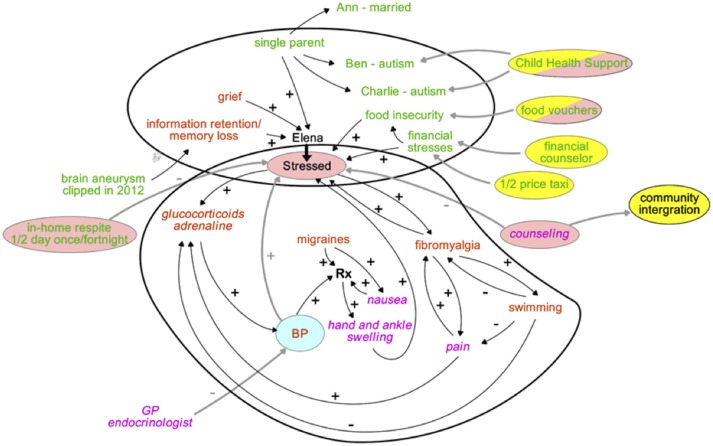
A system's map to appreciate the various dimensions affecting Elena's health journey, and service interventions to manage illness crises. Text colors indicate the nature of the key factors driving Elena's health, the bubble colors highlight the type of interventions aimed to restore her health. How to read the map: “+” indicates that a change of an item at the beginning of an arrow changes the item at the tip of the arrow in the same direction, “–” indicates that the change is in the opposite direction.

Successful management of Elena's multifaceted illness can be enhanced by drawing a systems diagram that communicates to all team members the various contributors and their interactions. Such understanding provides opportunities to not only make Elena aware of her triggers of health deterioration, but also allows the team to consider most likely beneficial interventions based on an appreciation of underlying social triggers and biological/physiological mechanisms (Figure 7) (38).

#### Miriam—Progressive Disease Deterioration With Unavoidable Admission?

**Figure 8:**
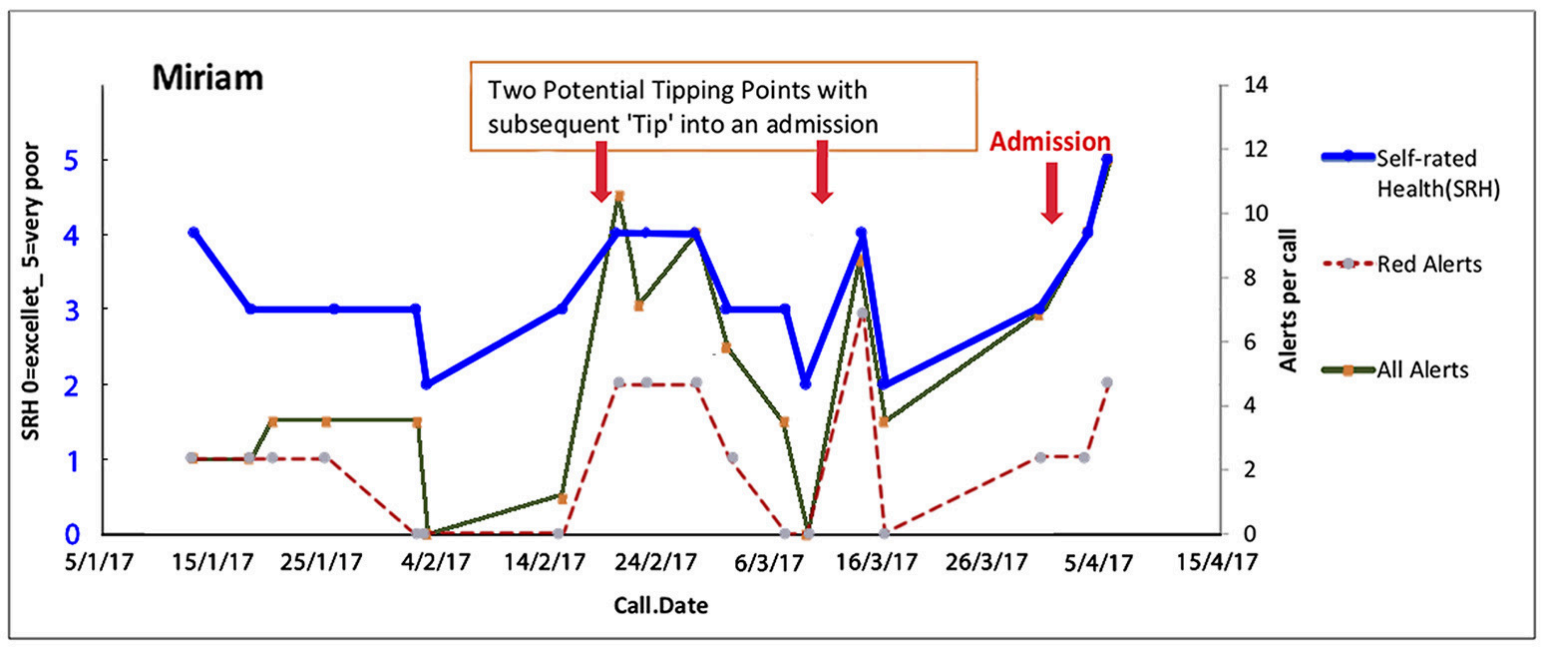
Miriam has unstable physical health with a short stay in hospital following a fall. Continuous support of her caregiver Peter is an essential element to maintain Miriam's health and independence at home. Here are two potential tipping points in Miriam's health (biopsychosocial) journey with frailty is unstable eventually leading to an admission. Trajectories of overall health today [Self-Related Health' (SRH−0 excellent, 1 very good, 2 good, 3 fair, 4 poor, 5 very poor). Red alerts are those pertaining to medical illness symptoms and total alerts refer to both medical and all alerts relate to wider biopsychosocial issues including coping, self-care, social, and environmental issues].

Miriam (a pseudonym) is an elderly lady who enjoys good health and is supported by her devoted son Peter (a pseudonym), who has difficulty recognizing and/or accepting the slow deterioration of his mother. While her health is generally stable, this segment of her journey represents a fall in which she sustained an injury which was unpredictable and resulted in an unavoidable short hospital stay for observation. Overall while she has very gradual increasing difficulties with balance, her general health, after a dip, continues on a stable trajectory (Figure 8).

The key anticipatory triggers in Miriam's trajectory are Peter's reluctance to acknowledge that his mother is increasingly frail. Peter was coached to accept the reality of his mother's potential for future instabilities and to trust the team. Peter was supported to provide accurate information rather than denying her subtle changes so that the team could intervene in time to possibly avert deterioration. This played an important contribution to anticipating and improving her trajectory.

### Summary of Process and Impact

Table 1 describes selected outcomes of the service deployment in MonashWatch.

An internal evaluation of MonashWatch is currently ongoing. Interim internal evaluation identified there has been an ~20–25% (= 1.8–2 days) reduction in hospital acute emergency bed days (LOS) for those in the Monash Watch program compared to usual care, based on the first 22 months of the current and ongoing trial. The reported reduction in hospital days is better than the target set (i.e., 10%) prior to the intervention. External evaluation of MonashWatch program as part of the wider HLCC program across Victoria is in progress.

Do patients value the program? To evaluate this question Monash Watch used a net-promoter scale, a tool to gauge the loyalty of a company's customers or clients. A net promoter score (NPS) can fall between −100 (i.e., where every customer as a “detractor” or would not recommend the product or service) and +100 (i.e., where every customer is a “promoter” or would recommend the product or service). In a net-promoter scale, clients are asked one question: “*would you recommend the service to family or colleague in a similar situation to yourself?*” and respond typically on a scale of one to ten.

A NPS is calculated by subtracting the percentage of customers who are detractors from the percentage of customers who are promoters. Generally, an NPS that is positive is considered to indicate loyalty, and an NPS above +50 is considered to reflect an excellent service or product (39). After 6-months of evaluation, 126 MW patients completed an anonymous net promoter scale, and 94% of participants reported that they would recommend the service to others, 5% were passives and 0% were detractors.

Currently, the cost per patient per year is ~$2,000 Australian based on a preliminary economic evaluation. This preliminary economic impact study suggested that a service with resource impacts of the order reported, would equate to very large cost savings over the remaining life of the cohort.

## Discussion

Many issues facing twenty-first century healthcare systems are no longer solvable with the prevailing mode of service organization and care delivery (6). Medical care in the first instance is about the person in front of us, their uniqueness is the result of the interplay between sociocultural context and biologically given blueprint (Sturmberg et al., submitted). These philosophical and scientific foundations provide the basis for redesigning care, in this case, the care for a group of patients defined by a high degree of vulnerability.

The key findings from this evaluation are that it is may be possible to sufficiently intervene in timely manner in some PPH trajectories at some junctures, such that there is a significant impact on a monitored cohort. Two markers of interoception—SRH and anticipated health over the next few days (AH), together with CG sense-making shifted significantly 3 days before an acute or emergency admission. This tipping pattern on around 3 days before an acute admission was in males and females and < 75 and 75+ with SRH being the most consistent marker CG anticipated deteriorations in males more than females in this small sample. Because frailty is a major predictor of admissions, it appears that interventions in psychosocial instabilities may be more fruitful than in interventions to delay admissions where the body is failing.

The PaJR approach demonstrates a means to better understand the underlying specific features in each person in the “PPH cohort,” and provides the insights needed to address each person's problems in the context of all their *particulars* (40). Each journey is unique; only respectful ongoing conversations will result in understanding each person and the life story of their health and illness pathway that allows for individualized support and intervention (consider Elena). PaJR in the context of MonashWatch and the rural Ireland pilot services (9) aims to avoid abstractions and embrace the all-important *individual* and *contextual* dimensions. Using lay people, i.e., non-health professionals from the local community, allows the de-medicalization of the health journey to the extent that the *particulars* of each individual are shared across a trusted team. In order to comply with ethical and safety concerns the non-professionals are trained in a variation of the community health worker model (41). This approach is disruptive to prevailing service delivery modes because data analytics and an expert PaJR decision support system are lifting trained non-professional to a higher level of function under the supervision of Health Coaches and other clinicians; this service structure allows conversations to remain focused on the *particulars* of this person. While disease management remains highly relevant, the whole person systemic view provides greater opportunities to enable individuals to self-care and achieve their life goals.

These findings fit well with Rosen's modeling relations and anticipations theories from several perspectives. Human health itself, Rosen saw as having two aspects, systemic and relational. Both components are necessary for the process of system description; they are: (a) a specification of what the system is like at any particular instant of time, and (b) a specification of how the system changes or will change state, as a function of present or past states and of the forces imposed on the system.

The specification of an appropriate set of variables to monitor in systemic and relational aspects is challenging.

Firstly, modeling highly appropriate and easily accessed metrics that mirror the real world human health state is essential. In this paper, we demonstrate that SRH, AH are useful markers in accordance with the emerging knowledge of personal interoception. Other metrics exist and more may emerge with the “internet of things,” however human conversations will always have an important role in understanding individual articulations of health concerns and external stressors. Accurate prediction is not necessarily the prime objective nor even possible in complex journeys, but trajectory analytics can assist anticipatory actions. Humans have highly capable anticipatory capability which can be tapped into via self-rated health. Other types of trajectory analytics may track disease development but taking account of interoception will be increasingly important as our knowledge develops.

Anticipatory care for people with complex trajectories, we argue, and this paper provides supporting evidence, should monitor perceived health current and anticipated future states that emerge from the dynamic network interactions between the microlevel of individual biology to the macrolevel factors of their environments. Clinical team functioning will be dependent upon the usefulness of their models or representations in the present environment and the future environment (35, 36). An anticipatory care provider/team has to talk about the current and the anticipated state to deal with the what is likely to become evident at a future point in time.

This paper is a present state analysis of theory, application, implementation of care related to a particular anticipatory care service in unstable health journeys. In accordance with Rosen's anticipatory model, it also aims to be an anticipation of how approaches will change, particularly in the understanding of interoception, as a function of present or past sensemaking, research and theories and of the forces imposed on health systems. None of this means reactive care is unimportant or avoidable, long-term preventative care remains the ideal.

## Conclusions

The MW service, incorporating PaJR, uses big data as the case finding mechanism using clinical algorithms from the public hospitals database in Victoria, Australia. Anticipatory care on a small local tailored data collection is supported by trajectory data analytics.

The internal evaluation of the PaJR system for patients with complex multiple morbidities and unstable health trajectories indicates that applying trajectory analytics, anticipatory and reactive care based upon scientific theories of interoception may result in effective and efficient health and social service delivery.

Big data may contain the context, but individual unstable journeys require human dialogue and sensemaking to anticipate the interoceptive timing and meaning behind potentially avoidable hospitalizations. Applying an anticipatory framework to appraise individual health in the context of healthcare services requires future data science strategies which are tailored to individual journeys, they most urgently need to incorporate human sense-making.

## Trial Registration

Retrospective Registration. MonashWatch is being independently externally evaluated with funding from the Department of Health and Human Services, Victoria, Australia.

## Author Contributions

CM initiated this paper and led revisions. JS, NH, KS, and DC contributed to the draft and revisions of the paper. CM reported data based upon the process and internal evaluation of the implementation of the PaJR system. All authors read and approve the final manuscript.

### Conflict of Interest Statement

CM is joint developer and owner of the PaJR software with KS. All evaluation of MonashWatch is carried out independently by Monash Health staff internally and externally by CSIRO. CM has no direct or commercial influence on outcomes of the evaluation. Process and formative evaluation is led by CM. The remaining authors declare that the research was conducted in the absence of any commercial or financial relationships that could be construed as a potential conflict of interest.

## References

[1] Yam CH Wong EL Chan FW Wong FY Leung MC Yeoh EK. Measuring and preventing potentially avoidable hospital readmissions: a review of the literature. Hong Kong Med J. (2010) 16:383–9. 20890004

[2] Martin C. Self-rated health: patterns in the journeys of patients with multi-morbidity and frailty. J Eval Clin Pract. (2014) 20:1010–6. 10.1111/jep.12133 24828245

[3] Martin CH Stockman N Campbell KD. Resilience, health perceptions, stressors and hospital admissions—observations from the real world of clinical care of unstable health journeys in Monash Watch (MW), Victoria, Australia. J Eval Clin Pract. (2018) 10.1111/jep.13031 30246430 PMC6283274

[4] McDaniel RR Jr. Lanham HJ Anderson RA. Implications of complex adaptive systems theory for the design of research on health care organizations. Health Care Manage Rev. (2009) 34:191–9. 10.1097/HMR.0b013e31819c8b38 19322050 PMC3667498

[5] Hudon C Chouinard MC Lambert M Diadiou F Bouliane D Beaudin J. Key factors of case management interventions for frequent users of healthcare services: a thematic analysis review. BMJ Open (2017) 7:e017762. 10.1136/bmjopen-2017-017762 29061623 PMC5665285

[6] Sturmberg JP Health System Redesign. How to Make Health Care Person-Centered, Equitable, and Sustainable. Cham: Springer (2018).

[7] Ferrier D Diver F Corin S McNair P Cheng C HealthLinks: incentivising better value chronic care in Victoria. Int J Integr Care (2017) 17:A129 10.5334/ijic.3241

[8] MonashWatch: Keeping People Healthy at Home Melbourne, VIC (2016). Available online at: http://monashhealth.org/page/monashwatch (Accessed August 19, 2018).

[9] Martin CM Vogel C Grady D Zarabzadeh A Hederman L Kellett J. Implementation of complex adaptive chronic care: the patient journey record system (PaJR). J Eval Clin Pract. (2012) 18:1226–34. 10.1111/j.1365-2753.2012.01880.x 22816797

[10] Rockwood K Stadnyk K MacKnight C McDowell I Hebert R Hogan D. A brief clinical instrument to classify frailty in elderly people. Lancet (1999) 353:205–6. 10.1016/S0140-6736(98)04402-X 9923878

[11] Vaishnavi S Connor K Davidson JR. An abbreviated version of the Connor-Davidson Resilience Scale (CD-RISC), the CD-RISC2: psychometric properties and applications in psychopharmacological trials. Psychiatry Res. (2007) 152:293–7. 10.1016/j.psychres.2007.01.006 17459488 PMC2041449

[12] Huling JD Yu M Liang M Smith M. Risk prediction for heterogeneous populations with application to hospital admission prediction. Biometrics (2018) 74:557–65. 10.1111/biom.12769 29073325 PMC6076875

[13] Martin CM Vogel C Hederman L Smith K Zarabzadeh A Grady D Avoidable hospitalizations in older adults. In: Sturmberg JP Martin CM editors. Handbook of Systems and Complexity in Health, New York, NY: Springer New York (2013). p. 445–65.

[14] Rosen R Anticipatory Systems: Philosophical, Mathematical, and Methodological Foundations. Oxford; New York, NY: Pergamon Press (1985).

[15] Dharmarajan K Hsieh AF Kulkarni VT Lin Z Ross JS Horwitz LI Trajectories of risk after hospitalization for heart failure, acute myocardial infarction, or pneumonia: retrospective cohort study. BMJ (2015) 350:h411 10.1136/bmj.h411 25656852 PMC4353309

[16] Krumholz HM. Post-hospital syndrome–an acquired, transient condition of generalized risk. N Engl J Med. (2013) 368:100–2. 10.1056/NEJMp1212324 23301730 PMC3688067

[17] Yu S Farooq F van Esbroeck A Fung G Anand V Krishnapuram B. Predicting readmission risk with institution-specific prediction models. Artif Intell Med. (2015) 65:89–96. 10.1016/j.artmed.2015.08.005 26363683

[18] Hollar D Trajectory Analysis in Health Care. Heidelberg: Springer (2018).

[19] Olde Rikkert MG Dakos V Buchman TG Boer R Glass L Cramer AO Slowing down of recovery as generic risk marker for acute severity transitions in chronic diseases. Crit Care Med. (2016) 44:601–6. 10.1097/CCM.0000000000001564 26765499

[20] Galvin R Gilleit Y Wallace E Cousins G Bolmer M Rainer T. Adverse outcomes in older adults attending emergency departments: a systematic review and meta-analysis of the identification of seniors at risk (ISAR) screening tool. Age Ageing (2017) 46:179–86. 10.1093/ageing/afw233 27989992

[21] Jylhä M. What is self-rated health and why does it predict mortality? Towards a unified conceptual model. Soc Sci Med. (2009) 69:307–16. 10.1016/j.socscimed.2009.05.013 19520474

[22] Li X Dunn J Salins D Zhou G Zhou W Schüssler-Fiorenza Rose SM. Digital health: tracking physiomes and activity using wearable biosensors reveals useful health-related information. PLoS Biol. (2017) 15:e2001402. 10.1371/journal.pbio.2001402 28081144 PMC5230763

[23] Phung TKT Siersma V Vogel A Waldorff FB Waldemar G. Self-rated versus caregiver-rated health for patients with mild dementia as predictors of patient mortality. Am J Geriatr Psychiatry (2018) 26:375–85. 10.1016/j.jagp.2017.06.005 28760512

[24] Martin C. What matters in “multimorbidity”? Arguably resilience and personal health experience are central to quality of life and optimizing survival. J Eval Clin Pract. (2018) 24:1282–4. 10.1111/jep.12644 27650998

[25] Shubin S Rapport F Seagrove A. Complex and dynamic times of being chronically ill: beyond disease trajectories of patients with ulcerative colitis. Soc Sci Med. (2015) 147:105–12. 10.1016/j.socscimed.2015.10.065 26560409

[26] Lyon ML. Psychoneuroimmunology: the problem of the situatedness of illness and the conceptualization of healing. Cult Med Psychiatry (1993) 17:77–97. 10.1007/BF01380601 8354075

[27] Damasio A Carvalho GB. The nature of feelings: evolutionary and neurobiological origins. Nat Rev Neurosci. (2013) 14:143–52. 10.1038/nrn3403 23329161

[28] Benyamini Y. Why does self-rated health predict mortality? An update on current knowledge and a research agenda for psychologists. Psychol Health (2011) 26:1407–13. 10.1080/08870446.2011.621703 22111660

[29] Craig AD. How do you feel? Interoception: the sense of the physiological condition of the body. Nat Rev Neurosci. (2002) 3:655–66. 10.1038/nrn894 12154366

[30] Kleckner IR Zhang J Touroutoglou A Chanes L Xia C Simmons WK. Evidence for a large-scale brain system supporting allostasis and interoception in humans. Nat Hum Behav. (2017) 1:0069. 10.1038/s41562-017-0069 28983518 PMC5624222

[31] Jylhä M Volpato S Guralnik JM. Self-rated health showed a graded association with frequently used biomarkers in a large population sample. J Clin Epidemiol. (2006) 59:465–71. 10.1016/j.jclinepi.2005.12.004 16632134

[32] Bennett JM Gillie BL Lindgren ME Fagundes CP Kiecolt-Glaser JK Inflammation through a psychoneuroimmunological lens. In: Sturmberg JP Martin CM editors. Handbook of Systems and Complexity in Health, New York, NY: Springer New York (2013). p. 279–99.

[33] Scheffer M Bolhuis JE Borsboom D Buchman TG Gijzel SMW Goulson D. Quantifying resilience of humans and other animals. Proc Natl Acad Sci USA. (2018) 115:11883–90. 10.1073/pnas.1810630115 30373844 PMC6255191

[34] Barrett LF Simmons WK. Interoceptive predictions in the brain. Nat Rev Neurosci. (2015) 16:419–29. 10.1038/nrn3950 26016744 PMC4731102

[35] Staiger TO Kritek PA Blakeney EL Zierler BK O'Brien K Ehrmantraut R A conceptual framework for applying the anticipatory theory of complex systems to improve safety and quality in healthcare. In: Nadin M editor. Anticipation and Medicine, Heidelberg: Springer (2016). p. 31–40.

[36] Pinaire J Azé J Bringay S Landais P. Patient healthcare trajectory. An essential monitoring tool: a systematic review. Health Inf Sci Syst. (2017) 5:1. 10.1007/s13755-017-0020-2 28413630 PMC5390363

[37] Martin C Sturmberg JP Stockman K Campbell D Hederman L Vogel C Supporting complex dynamic health journeys using conversation to avert hospital readmissions from the community: an ecological perspective incorporating interoception. In: Sturmberg J editor. Putting Systems and Complexity Science into Practice, Cham: Springer (2018).

[38] Sturmberg JP. Systems and complexity thinking in general practice. Part 1—clinical application. Aust Fam Physician (2007) 36:170–3. 17339983

[39] Reichheld F Markey R The Ultimate Question 2.0: How Net Promoter Companies Thrive in a Customer-Driven World. Boston, MA: Harvard Business Review Press (2011). p52.

[40] McWhinney IR ‘An acquaintance with particulars…'. Fam Med. (1989) 21:296–8. 2753257

[41] Kwan BM Rockwood A Bandle B Fernald D Hamer MK Capp R. Community health workers: addressing client objectives among frequent emergency department users. J Public Health Manag Pract. (2018) 24:146–54. 10.1097/PHH.0000000000000540 28141671 PMC5794249

